# Supplementary material for: How to Make an Internal Team Coach: An Integration of Research
Source: Behav Sci (Basel). 2024 May 27;14(6):452. doi: 10.3390/bs14060452 (PMC11200866; doi:10.3390/bs14060452)
Supplement: Supplementary file 1 [file behavsci-14-00452-s001.zip › behavsci-2952532-supplementary.pdf]

Table S1.

Papers included in review (N = 262)

#	Authors	Title	Notes	Themes exhibited
1	Head et al. (2022)	"Opening eyes to real interprofessional education": Results of a national faculty development initiative focused on interprofessional education in oncology palliative care.	• Discusses how the program left participants with improved teamwork and collaboration skills, utilized a training program and a written reflection.	• Engages in reflective practice.
2	Conger (1993)	Personal growth training: Snake oil or pathway to leadership?	• Discusses the need to know oneself and how this can empower participants to take accountability.	• Allows autonomy.<br>• Acknowledges and is aware of their/team needs.
3	Liang et al. (2023)	Physician perceptions of administrative leadership development post-COVID-19	• Discusses how physicians sought out development as a reaction rather than doing it proactively.	• Acknowledges and is aware of their/team needs.
4	Main et al. (2022)	Preparing industry leaders: The role of doctoral education and early career management training in the leadership trajectories of women STEM PhDs	• Discusses the human capital theory and how knowledge increases one's human capital.	• Engages in reflective practice.
5	Brandstorp et al. (2016)	Primary care emergency team training in situ means learning in real context.	• Discusses how participants had consistent long-term motivation to strengthen safety and maintain team training.	• Engages in reflective practice.
6	Brown (2023)	Profiling leadership: Attitudes, knowledge and training in the biological sciences	• Discusses how participants believe they demonstrate effective leadership, participants believed feedback was important.<br>• Emphasizes the importance of making learning a priority.	• Engages in reflective practice.
7	Grøn et al. (2020)	Public managers' leadership identity:	• Discusses how leaders need to recognize	• Initiates structure.

		Concept, causes, and consequences	responsibility and be goal-oriented.	
8	Blakeney et al. (2019)	Purposeful interprofessional team intervention improves relational coordination among advanced heart failure care teams	• Discusses how a lack of collective understanding can be linked to only a small modest increase in shared knowledge.	• Initiates structure.<br>• Organizes team information.
9	Lachance and Oxendine (2015)	Redefining leadership education in graduate public health programs: Prioritization, focus, and guiding principles	• Discusses the motivation team members already have and the importance of problem identification.	• Initiates structure.
10	McGaghie et al. (2010)	A critical review of simulation-based medical education research	• Discusses the importance of feedback, deliberate practice, and actually measuring outcomes of simulation training.	• Engages in reflective practice.
11	Figueiredo et al. (2022)	A decade of research on leadership and its effects on creativity-innovation: A systematic and narrative literature review.	• Discusses the importance of shared goals and team cooperative norms as well as facilitating the role of psychological safety.	• Initiates structure.<br>• Maintains interpersonal sensitivity.<br>• Facilitates information sharing.
12	Thanh et al. (2021)	A framework of leadership and managerial competency for preventive health managers in Vietnam.	• Discusses the importance of emotional intelligence for motivation.	• Maintains interpersonal sensitivity.
13	Szelwach et al. (2023)	A holistic approach to embodied leadership development at the US Coast Guard Academy	• Emphasizes the importance of psychological safety in order to reduce fear and create opportunities for difficult conversations from different perspectives.	• Engages in open dialogue.<br>• Facilitates information sharing.
14	Aaberg et al. (2021)	A human factors intervention in a hospital - Evaluating the outcome of a TeamSTEPPS program in a surgical ward.	• Discusses transfer of training and the importance of internal and external environments for learning.	• Acknowledges and is aware of their/team needs.

15	Lyons (2007)	A leadership development model to improve organizational competitiveness.	• Discusses expectations around follower performance and how this can affect outcomes.	• Initiates structure.<br>• Allows autonomy.
16	Goski et al. (2002)	A model of leadership development.	• Discussed a development program that involved activities like reflecting on one's role and how to build critical relationships.	• Maintains interpersonal sensitivity.<br>• Engages in reflective practice.
17	Lee et al. (2014)	A multiple group analysis of the training transfer model: Exploring the differences between high and low performers in a Korean insurance company.	• Discusses how one's environment can affect knowledge transfer and how self-esteem and self-efficacy can lead to greater performance.	• Acknowledges and is aware of their/team needs.<br>• Facilitates information sharing.
18	Körner et al. (2018)	A patient-centered team-coaching concept for medical rehabilitation.	• Discusses goal setting, making goals clear in order to make knowledge integration better.<br>• Emphasizes a needs-analysis approach.	• Initiates structure.<br>• Allows autonomy.<br>• Offer support.
19	Sethi et al. (2021)	A pilot study of the implementation and evaluation of a leadership program for medical undergraduate students: Lessons learned	• Emphasizes how students with specific interests were the ones that could make changes in organizational practices.	• Initiates structure.
20	Siengthai (2015)	A reflection on a leadership training programme of the ministry of interior, Thailand	• Discusses pre- and post-existing participant motivation that is actively affected by environmental favorability.	• Acknowledges and is aware of their/team needs.<br>• Facilitates information sharing.
21	Corrigan et al. (2000)	A short course in leadership skills for	• Discusses learning activities consisting of	• Engages in

		the rehabilitation team	lectures, role play, and active problem solving.	reflective practice.
22	Bhrawni et al. (2017)	A stakeholder-based approach to leadership development training: The case of medical education in Canada	● Emphasizes the need for interpersonal and strategic skills for strong leadership, as well as a thoughtful curriculum that considers these factors.	● Maintains interpersonal sensitivity.
23	Klaber et al. (2008)	A structured approach to planning a workbased leadership development programme for doctors in training.	● Conducts an analysis akin to a needs-analysis prior to installment and the importance of program evaluation.	● Engages in reflective practice.
24	Dalgaard et al. (2023)	A study protocol outlining the development and evaluation of a training program for frontline managers on leading well-being and the psychosocial work environment in Danish hospital settings – a cluster randomized waitlist controlled trial.	● Emphasizes the need for managers to exhibit self-care so that their team also has self-care and psychological safety.<br>● Training needs to involve the well-being of managers as well.	● Acknowledges and is aware of their/team needs.
25	Thoms and Greenverger (1998)	A test of vision training and potential antecedents to leaders' visioning ability	● Discusses how personality and individual differences affect visioning activity.	● Acknowledges and is aware of their/team needs.
26	McElroy and Stark (1992)	A thematic approach to leadership training	● Discusses a variety of general leadership skills and training themes, such as diagnosis, communication, negotiation, and goal setting, among others.	● Initiates structure.<br>● Offers support.<br>● Engages in open dialogue.
27	Hackman and Wageman (2005)	A theory of team coaching.	● Discusses how coaching works from beginning to end, and how the beginning is important to offer teams a good way to orient the task.	● Initiates structure.
28	Newsom and Dent (2010)	A work behavior analysis of executive coaches	● Discusses goal setting and identifying coaching goals to find organizational alignment, as well as importance of interpersonal skills.	● Initiates structure<br>● Maintains interpersonal sensitivity.

29	Alzen et al. (2021)	Academic coaching and its relationship to student performance, retention, and credit completion	• Discusses goal setting and emphasizes rapport for performance.	• Initiates structure.<br>• Facilitates information sharing.
30	Söderhjelm et al. (2018)	Academic leadership: management of groups or leadership of teams? A multiple-case study on designing and implementing a team-based development programme for academic leadership.	• Discusses how the program affected knowledge transfer.	• Facilitates information sharing.
31	Backus et al. (2010)	Accelerating leadership development via immersive learning and cognitive apprenticeship	• Discusses immersive learning and how knowledge transfer occurs via job-related activities.	• Organizes team information.<br>• Engages in reflective practice.
32	Mulec and Roth (2005)	Action, reflection, and learning – Coaching in order to enhance the performance of drug development project management teams.	• Discusses how coaches can help teams by processing inside knowledge of procedures and politics.	• Acknowledges and is aware of their/team needs.<br>• Organizes team information.
33	Patwardhan et al. (2022)	Ad agency leadership in the US, UK, and Australia: A mixed-method analysis of effective attributes and styles. Journal of advertising, 51(2), 223-239.	• Emphasizes the importance of soft skills for great leadership.	• Maintains interpersonal sensitivity.
34	Hirschfeld and Thomas (2011)	Age-and gender-based role incongruence: Implications for knowledge mastery and observed leadership potential among personnel in a leadership development program.	• Discusses how knowledge mastery is important for evaluation in terms of interpersonal bias.	• Maintains interpersonal sensitivity.
35	Mohammed et al.	An empirical study of the relationship	• Discusses creating a motivational climate to	• Initiates structure.

	(2023)	between leadership practice in training programs and skill development: Motivational climate as mediating	increase performance.	
36	Stephens et al. (2016)	An interprofessional training course in crises and human factors for perioperative teams	• Discusses a program focused on crises and human factors, with learning goals such as appreciating the elements of effective teamwork and the importance of reviewing after an incident.	• Engages in reflective practice.
37	Hasson et al. (2016)	Are we all in the same boat? The role of perceptual distance in organizational health interventions	• Discusses an intervention that consists of a theoretical and practical part, focusing on feedback opportunities and practice.	• Initiates structure.<br>• Engages in reflective practice.
38	Zammuner et al. (2013)	Assessing and Training Leaders' Emotional Intelligence, and Testing its Influence on Leaders' Employees.	• Emphasizes having concern for interpersonal behavior and the importance of emotional skills.	• Maintains interpersonal sensitivity.
39	May et al. (2019)	Assessment of leadership behavior in occupational health and safety.	• Discusses how goals can be used to monitor safety performance and how the coach influences how team members execute goals.	• Initiates structure.
40	Ginzburg et al. (2018)	Assessment of medical students' leadership traits in a problem/case-based learning program.	• Discusses individual differences and assessing one's own progress as well as getting feedback from facilitators.	• Engages in reflective practice.
41	Fouilloux et al. (2014)	Assessment of team training in management of adverse acute events occurring during cardiopulmonary bypass procedure: A pilot study based on an animal simulation model.	• Discusses a simulation model that also involves debriefs.	• Engages in reflective practice.
42	Skipper and Bell (2006)	Assessment with 360 evaluations of leadership behavior in construction project managers	• Discusses feedback via 360-degree evaluations.	• Engages in reflective practice.
43	Neily et al. (2010)	Association between implementation	• Emphasizes how the briefing and debriefing	• Engages in

		of a medical team training program and surgical mortality	process became more comprehensive after training and how this can help improve performance, in this case tied to patient outcomes.	reflective practice.
44	Fusco et al. (2015)	Authentic leaders are... conscious, competent, confident, and congruent: A grounded theory of group coaching and authentic leadership development.	● Includes articulating a compelling vision for the future and providing employees with support.<br>● Discusses listening to others and helping them understand their own leadership styles and experiences.<br>● Discusses developing participants' own self-knowledge.	● Initiates structure.<br>● Offers support.<br>● Acknowledges and is aware of their/team needs.<br>● Organizes team information.
45	Mao et al. (2023)	Authentic leadership and employee resilience during the COVID-19: The role of flow, organizational identification, and trust.	● Discusses flow, authentic leadership, and having clear goals.	● Initiates structure.<br>● Acknowledges and is aware of their/team needs.
46	Hirschfeld et al. (2006)	Becoming team players: Team members' mastery of teamwork knowledge as a predictor of team task proficiency and observed teamwork effectiveness.	● Emphasizes the importance of teamwork skills for task performance.	● Maintains interpersonal sensitivity.
47	Rhodes and Martin (2014)	Behavior change after adventure education courses: Do work colleagues notice?	● Discusses how the course can help participants deal with challenges and have a more positive attitude.	● Offers support.
48	Holmemo et al. (2023)	Beyond the lean manager: Insights on how to develop corporate lean leadership.	● Discusses the importance of creating vision and aligning team goals.	● Initiates structure.
49	Hollenbeck et al. (2004)	Bridging the gap between I/O research and HR practice: Improving team composition, team training, and team task design	● Discusses goal setting and the importance of interpersonal KSAOs, such as collaborative problem solving.<br>● Emphasizes the need for team leader support and establishing shared mental models.	● Initiates structure.<br>● Acknowledges and is aware of their/team needs.<br>● Engages in open

				dialogue.<br>● Organizes team information.
50	Widdowson et al. (2020)	Bridging the team coaching competency gap: A review of the literature.	● Discusses the need to be a skilled facilitator to help the team complete their goals.<br>● Discusses the four types of knowledge a team coach needs to have, in everything from understanding group dynamics to context.	● Organizes team information.<br>● Facilitates information sharing.
51	Mosson et al. (2019)	Building implementation capacity (BIC): A longitudinal mixed methods evaluation of a team intervention	● Emphasizes how implementation is a team effort and how to combine knowledge through shared experiences.	● Organizes team information.
52	Hill and Stephens (2005)	Building leadership capacity in medical education: Developing the potential of course coordinators	● Discusses the development of a leadership course with factors such as cultivating conditions for change.	● Facilitates information sharing.
53	Pledger (2007)	Building manager effectiveness by combining leadership training and organization development.	● Discusses how the individuals should assess leadership style and motivation triggers.	● Acknowledges and is aware of their/team needs.
54	Hultgren et al. (2022)	Can cognitive behavioral team coaching increase well-being?	● Providing a structure for the team is necessary, setting realistic goals is vital.<br>● Building openness and trust is important, emphasizing the need for time management for coaching.<br>● By sharing and discussing ideas together, knowledge integration can be a smoother process.	● Initiates structure.<br>● Offers support.<br>● Organizes team information.
55	Henriksen and Borgesen (2016)	Can good leadership be learned through business games?	● Discusses how games can help improve leadership performance.	● Engages in reflective practice.
56	Jacobsen et al. (2022)	Can leadership training improve organizational effectiveness? Evidence from a randomized field experiment	● Discusses transformational leadership and using verbal awards.	● Acknowledges and is aware of their/team needs.

		on transformational and transactional leadership		
57	O'Conner and Menaker (2008)	Can massively multiplayer online gaming environments support team training?	• Emphasizes how games can help promote learning and have the potential to help individuals reflect and be immersed in situations, as well as practice with feedback.	• Engages in reflective practice.
58	Van Schaik et al. (2015)	Challenges of interprofessional team training: A qualitative analysis of residents' perceptions	• Emphasizes the importance of psychological safety for training.	• Facilitates information sharing.
59	Kendra and Taplin (2004)	Change agent competencies for information technology project managers.	• Discusses competencies needed for project managers such as communication, teamwork, and continuous learning, among others.	• Maintains interpersonal sensitivity.
60	Weller et al. (2012)	Change in attitudes and performance of critical care teams after a multi-disciplinary simulation-based intervention.	• Discusses how an intervention helped doctors be better able to assign team roles and use closed-loop communication.	• Initiates structure.<br>• Engages in open dialogue.
61	Hernandez and Longman (2020)	Changing the face of leadership in higher education: "Sponsorship" as a strategy to prepare emerging leaders of color.	• Discusses how the program awakened awareness of the need for mentors and sponsors, the need for clear language, and how empowerment can help navigate career advancement.	• Acknowledges and is aware of their/team needs.
62	Bateman and King (2020)	Change nurse leadership training comparison: Effective and timely delivery	• Discusses blended learning for training but also a need to measure changes in outcomes.	• Engages in reflective practice.
63	Darby (2022)	Co-creating online leadership learning spaces: Emerging practices in graduate and professional leadership education.	• Discusses how to improve online training with things such as group projects, informal interactions, and being responsive to deadlines as well as giving feedback.	• Acknowledges and is aware of their/team needs.<br>• Engages in reflective practice.

64	Couch and Rose (2020)	Coaching culturally different members of international business teams-the role of cultural intelligence	• Emphasizes the need for cultural sensitivity when coaching international teams.	• Maintains interpersonal sensitivity.
65	Reich et al. (2009)	Coaching product development teams: A conceptual foundation for empirical studies.	• Emphasizes the need to develop a common tongue.	• Organizes team information.
66	Okpala et al. (2021)	Collective transformation: Outcomes of coaching as part of a leadership development program for STEM fellows at historically Black colleges and universities	• Discusses how coaching can help inspire and motivate professional growth, and help people develop intentional reflection and a success-orientation, among other characteristics.	• Offers support.<br>• Engages in reflective practice.
67	Getha-Taylor et al. (2015)	Considering the Effects of Time on Leadership Development.	• Discusses how training effects can vary and how understanding this delay can help better plan training activities.	• Engages in reflective practice.
68	Wiessner and Gonzalez Sullivan (2007)	New learning: Constructing knowledge in leadership training programs.	• Discusses knowledge construction and concepts such as reframing, embracing, and embodying activities.	• Organizes team information.
69	O'Neill et al. (2017)	Constructive controversy and reflexivity training promotes effective conflict profiles and team functioning in student learning teams	• Discusses constructive conflict and how this can maybe help team performance by increasing innovation.	• Maintains interpersonal sensitivity.<br>• Facilitates information sharing.
70	Ray et al. (2017)	Creating a sustainable advantage through team building.	• Discusses making a team charter at large and having open conversations to achieve better team performance.	• Initiates structure.<br>• Engages in open dialogue.
71	Balwant (2021)	Crisis leadership: Teaching external corporate communications via an experiential learning exercise.	• Discusses using a simulation technique to help students understand how to deal with external corporate communications.	• Engages in reflective practice.

72	Johnson et al. (2023)	Designing a leadership and management training curriculum for undergraduate health professions students: Lessons from the University of Sierra Leone.	• Discusses a training program that incorporated a variety of strategies such as group sessions mentoring, and peer reflections.	• Engages in reflective practice.
73	Nafukho et al. (2016)	Developing emotional intelligence skills among practicing leaders: reality or Myth?	• Discusses a program to aid leaders in increasing their emotional intelligence.	• Maintains interpersonal sensitivity.
74	Tsoh et al. (2019)	Developing faculty leadership from 'within': A12-year reflection from an internal faculty leadership development program of an academic health sciences center.	• Discusses the development of a training program and remarks on the need for booster sessions and additional support for leaders	• Offers support.<br>• Engages in reflective practice.
75	Barbaroux (2022)	Developing leadership skills through simulation-based training: A research framework and interpretive case study	• Discusses a training session that involves three main parts: action, planning, debriefing.<br>• Emphasizes discussing goals and reflecting on lessons learned.	• Initiates structure.<br>• Engages in reflective practice.
76	Bickle (2017)	Developing remote training consultants as leaders—Dialogic/network application of path-goal leadership theory in leadership development	• Discusses path–goal theory and how leaders can use this for leadership development, such as being directive, supportive, participative, and more.	• Initiates structure.<br>• Offers support.
77	Pless et al. (2011)	Developing responsible global leaders through international service-learning programs: The Ulysses experience	• Discusses a variety of competencies, such as responsible leadership competencies and global leadership competencies.<br>• Emphasized cultural intelligence and maintaining a global mindset.	• Maintains interpersonal sensitivity.
78	Neill and de Klerk (2023)	Developing the soul framework to enhance employee experience in teams as part of a team coaching approach.	• Discusses the SOUL team coaching framework which includes four phases: settle, offer, unite, and learn.	• Initiates structure.

79	Wang et al. (2008)	Development and evaluation of a leadership training program for public health emergency response: Results from a Chinese study.	• Describes an emergency training program that involved learning objectives such as identifying issues, evaluating them, and then applying problem solving skills.	• Initiates structure.
80	Liu et al. (2009)	Disseminating the functions of team coaching regarding research and development team effectiveness: Evidence from high-tech industries in Taiwan	• Discusses a variety of behaviors, such as structuring the team, establishing team goals, and arranging resources required to accomplish the team tasks.<br>• Leaders need to remove organizational impediments and help individual members strengthen their own personal contributions.<br>• The use of appropriate strategies is important.	• Initiates structure.<br>• Allows autonomy.<br>• Offers support.<br>• Acknowledges and is aware of their/team needs.<br>• Organizes team information.
81	Salas et al. (1999)	Does CRM training improve teamwork skills in the cockpit?: Two evaluation studies	• Discusses evaluation of a training that involved requesting better communication, analyzing multiple issues at hand, and more.	• Acknowledges and is aware of their/team needs.<br>• Engages in open dialogue.
82	Salas et al. (2008)	Does team training improve team performance? A meta-analysis	• Presents meta-analytic evidence supporting the idea that team training works, with team size and training content may moderate these effects.	• Acknowledges and is aware of their/team needs.
83	Warner (2012)	E-coaching systems: Convenient, anytime, anywhere, and nonhuman.	• Presents the idea that coaching can be performed virtually and how they can help the client track goal process.	• Initiates structure.
84	Barling et al. (1996)	Effects of transformational leadership training on attitudinal and financial outcomes: A field experiment	• Focuses on transformational leadership and how this can help managers have more charisma than those in the non-control group, as well as more individual consideration.	• Offers support.
85	Gjerra et al. (2014)	Efficacy of simulation-based trauma team training of non-technical skills. A systematic review.	• Analyzes studies and finds that simulation-based training did improve performance but also touched on how it can be difficult to	• Engages in reflective practice.

			maintain these effects.	
86	Chen and Hix (2022)	Embracing changes: Virtual change management and leadership training implications	• Discusses how training can help managers adapt to external circumstances and develop changing mindsets.	• Acknowledges and is aware of their/team needs.
87	Khalid et al. (2021)	Empowering women at the higher institutional level: analysis of business education and leadership training program.	• Emphasizes the importance of self-reflection for leadership capacity.	• Acknowledges and is aware of their/team needs.<br>• Engages in reflective practice.
88	Lonka et al. (2019)	Engaging leadership training–fostering social interaction skills through e-learning and blended solutions	• Discusses how this training improves communication skills.	• Engages in open dialogue.
89	Samuel and Durning (2022)	Enhancing leadership training through an experiential approach: An online model for the 21st century	• Discusses how coaches can help individuals develop learning agreements, and touches on debriefing.	• Initiates structure.<br>• Engages in reflective practice.
90	Stout et al. (1997)	Enhancing teamwork in complex environments through team training	• Emphasizes the importance of teamwork skills for team performance, such as communication, assertiveness, and situational awareness.	• Engages in open dialogue.<br>• Acknowledges and is aware of their/team needs.
91	Kirkman et al. (2006)	Enhancing the transfer of computer-assisted training proficiency in geographically distributed teams.	• Emphasizes the importance of team trust (among other variables) for training proficiency.	• Facilitates information sharing.
92	Rapp et al. (2007)	Evaluating an individually self-administered generic teamwork skills training program across time and	• Developing team charters and determining member roles is important.<br>• Discusses facilitating team interactions, building	• Initiates structure.<br>• Facilitates information

		levels.	a collaborative environment, and team problem solving.<br>• Discusses team training and simulation.	sharing.
93	Clark (2002)	Evaluating an interdisciplinary team training institute in geriatrics: Implications for teaching teamwork theory and practice	• Discusses how focusing on relevant knowledge is vital, as well as using a blended learning approach to improve training.	• Allows autonomy.
94	Kass and Grandzol (2012)	Evaluating the value-added impact of outdoor management training for leadership development in an MBA program.	• Leaders need to be motivated for their team.<br>• Discusses emotional intelligence at large, such as self-awareness and management of emotion.<br>• Discusses how knowledge is created via experiencing, reflecting, thinking, and acting.<br>• Training included reflective observation and active experimentation (akin to simulation).	• Acknowledges and is aware of their/team needs.<br>• Organizes team information.<br>• Engages in reflective practice.
95	Seeg et al. (2022)	Explaining and enhancing training transfer: A consumer-centric evaluation of a leadership training.	• Discusses SMART Goals.<br>• Discusses interpersonal competencies such as emotion management, as well as the need to give feedback and appraisal.<br>• The training session utilized included a booster session.	• Initiates structure.<br>• Maintains interpersonal sensitivity.<br>• Engages in reflective practice.
96	Allen and Shehane (2016)	Exploring the language of leadership learning and education	• Discusses language to use for crafting common leadership language in an organization, such as defining the context, having an inclusive approach, garnering support, and more.	• Acknowledges and is aware of their/team needs.
97	van Dorssen-Boog et al. (2021)	Facilitating health care workers' self-determination: The impact of a self-leadership intervention on work engagement, health, and performance	• Emphasizes the importance of work engagement for self-leadership.	• Allows autonomy.
98	Rentsch et al. (2010)	Facilitating knowledge building in teams: Can a new team training	• Discusses how to build knowledge in teams.	• Organizes team information.

		strategy help?		• Facilitates information sharing.
99	Grantham et al. (2014)	Female graduate students' attitudes after leadership training: A case study	• Discusses an intervention that involved networking, goal setting, skills training, and mentoring.	• Initiates structure.
100	Yayac et al. (2019)	Formal leadership training for orthopedic surgeons: Limited opportunities amongst growing demand.	• Discusses the need for more leadership training for orthopedic surgeons as it is necessary given their environment.<br>• Effective training should include information on communication and team building, among others.	• Engages in open dialogue.
101	Ewert and Overholt (2010)	Fostering leadership through a three-week experience: Does outdoor education make a difference?	• Discusses an outdoor education program that involved developing others, developing oneself, and assessing the environment, among others.	• Acknowledges and is aware of their/team needs.<br>• Maintains interpersonal sensitivity.
102	Lüchinger et al. (2023)	French-speaking Swiss physician's perceptions and perspectives regarding their competencies and training need in leadership and management: A mixed-methods study	• Emphasizes how physicians want training that will help them manage teams and manage conflict.	• Maintains interpersonal sensitivity.
103	Hurley and Ketai (1993)	From small-group members to leaders: Conflicting changes in behavioral ratings given and received.	• Discusses how these ratings may improve interpersonal skills.	• Maintains interpersonal sensitivity.
104	Sims et al. (2022)	Furthering women faculty in leadership roles: A human performance improvement case study	• Discusses logic model that touches on a variety of leadership points, emphasizes goals.	• Initiates structure.
105	Ali and Knox (2021)	Gaming leadership development with multi-rater assessment	• Discusses leadership competencies in the gaming industry, such as creativity, innovation,	• Maintains interpersonal

			communication, and relationship (i.e., ability to maintain social ties within gaming community).	sensitivity.
106	Raudenbush and Marquardt (2008)	Growing leaders at the US Department of Agriculture: A case study of leadership development using action learning	• Discusses interpersonal and communication skill competencies.	• Maintains interpersonal sensitivity.
107	Black and Gregersen (2000)	High impact training: Forging leaders for the global frontier	• Discusses a model of global leadership training that involves mental remapping.	• Organizes team information.
108	Woodhead (2019)	How does coaching help to support team working? A case study in the NHS.	• Coaching needs to focus on clarifying shared goals, the team needs to have commitment and sustainability.<br>• It is important to foster a safe place for opening up, as well as a place where information can "cascade."	• Initiates structure.<br>• Offers support.<br>• Engages in open dialogue.<br>• Organizes team information.
109	Rajasinghe and Garvey (2023)	How experiencing executive coaching helps coaches feel they are independent learners and self-coaches: An interpretative phenomenological analysis.	• Discusses distinct models of learning but emphasizes how coaching can help people become independent learners.	• Allows autonomy.
110	Wageman (2001)	How leaders foster self-managing team effectiveness: Design choices versus hands-on coaching	• Discusses stretch goals.<br>• Emphasizes the importance of being able to work through interpersonal issues.	• Initiates structure.<br>• Maintains interpersonal sensitivity.
111	Clarke and Higgs (2016)	How strategic focus relates to the delivery of leadership training and development	• Discusses the importance of engaging staff for motivation.	• Offers support.
112	De Brún and McAuliffe	Identifying the context, mechanisms and outcomes underlying collective leadership in teams: Building a realist programme theory.	• Discusses encouraging the team to self-manage and use co-design for improvement.<br>• Emphasizes the need for open, regular, and inclusive communication as well as shared	• Allows autonomy.<br>• Engages in open dialogue.

			responsibility for the team's performance.	
113	Richter et al. (2015)	iLead-a transformational leadership intervention to train healthcare managers' implementation leadership.	• Discusses the importance of feedback for managers to improve in their leadership skills, presents a generic leadership training.	• Engages in reflective practice.
114	Fung et al. (2015)	Impact of crisis resource management simulation-based training for interprofessional and interdisciplinary teams: A systematic review	• Discusses the implications of CRM and simulation for interdisciplinary team and presents evidence suggesting its benefits.	• Engages in reflective practice.
115	Hopkins et al. (2022)	Impact of leadership development and facilitated peer coaching on women's individual, collective, and organizational behaviors in human services.	• Discusses goal setting and adjusting goals according to environment.<br>• Discusses collective sharing of knowledge.	• Initiates structure.<br>• Engages in open dialogue.
116	Krejci and Mallin (1997)	Impact of leadership development on competencies.	• Discusses developing leadership competencies through knowledge-based training.<br>• Focusses on team learning.	• Organizes team information.
117	Willgerodt et al.	Impact of leadership development workshops in facilitating team-based practice transformation.	• Discusses shared mental models and the need for closed-loop communication as well as having all members of the team being actively involved in the task.<br>• Emphasizes the need for interpersonal and reflective skills.	• Engages in open dialogue.<br>• Facilitates information sharing.<br>• Maintains interpersonal sensitivity.
118	Dvir et al. (2002)	Impact of transformational leadership on follower development and performance: A field experiment	• Discusses goal setting and focusing on the goals of the followers as well as leaders' own.	• Allows autonomy.
119	Tahtali et al. (2017)	Implementation of stroke teams and simulation training shortened process times in a regional stroke network—A network-wide prospective trial.	• Discusses simulation-based training and the importance of sharing knowledge among team members for better performance.	• Engages in open dialogue.<br>• Facilitates information

				sharing.
120	Kyrkjebø et al. (2006)	Improving patient safety by using interprofessional simulation training in health professional education.	• Discusses involving students in interprofessional training to enhance the learning processes of students through reflection and other activities.	• Allows autonomy.<br>• Engages in reflective practice.
121	Nielsen et al. (2022)	In the eye of the beholder: How self-other agreements influence leadership training outcomes as perceived by leaders and their followers	• Discusses goal setting to improve strengths and notes importance of realizing weaknesses.	• Initiates structure.<br>• Acknowledges and is aware of their/team needs.
122	Siaglet et al. (2015)	Insight into team competence in medical, nursing and respiratory therapy students	• Discusses simulation-based training to learn and increase fundamental knowledge .	• Engages in reflective practice.
123	Zenger and Folkman (2013)	Inspiring & motivating to achieve top performance	• Discusses goal setting.	• Initiates structure.
124	Farrell (2016)	Interim leadership	• Discusses the importance of specifying goals.	• Initiates structure.
125	Lim et al. (2021)	Interpretive leadership skill in meaning-making by nonprofit leaders.	• Discusses goal setting and importance of aligning personal value/efforts with organizational goals.	• Initiates structure.<br>• Allows autonomy.
126	Ericson et al. (2012)	Interprofessional clinical training for undergraduate students in an emergency department setting.	• Discusses how learning can be facilitated by the environment.<br>• Emphasizes the importance of knowing one’s and others’ competencies.	• Acknowledges and is aware of their/team needs.<br>• Facilitates information sharing.
127	Kiessling et al. (2022)	Interprofessional simulation-based team-training and self-efficacy in emergency medicine situations.	• Emphasizes importance of feedback.	• Engages in reflective practice.

128	Giannitrapani et al. (2018)	Interprofessional training and team function in patient-centered medical home: Findings from a mixed method study of interdisciplinary provider perspectives.	• Discusses creating a positive learning environment and providing knowledge and skills to those who may lack them.	• Offers support.<br>• Facilitates information sharing.
129	Facca-Miess (2015)	Investigating teaching leadership in the capstone marketing course	• Discusses goal setting.	• Initiates structure.
130	Rios and Nogueira (2023)	Knowledge of the nursing team on cardiopulmonary resuscitation before and after training	• Emphasizes importance of knowing one's job domain.	• Acknowledges and is aware of their/team needs.
131	Hurwitz and Hurwitz (2023)	Leadership and followership training: Everyone, everywhere, all at once	• Discusses the importance of building interpersonal relationships.	• Maintains interpersonal sensitivity.
132	Hemmer et al.	Leadership and management training for residents and fellows: A curriculum for future medical directors.	• Emphasizes the need for interpersonal skills with an emphasis on time and change.	• Maintains interpersonal sensitivity.
133	Waldman et al. (2011)	Leadership and neuroscience: Can we revolutionize the way that inspirational leaders are identified and developed?	• Discusses vision of the leader and a focus on the goals of the collective.	• Initiates structure.<br>• Offers support.
134	Wang et al. (2023)	Leadership competencies in the financial Industry during digital transformation: An evaluation framework using the Z-DEMATEL Technique	• Discusses allotting structure and authority to achieve goals.<br>• Emphasizes interpersonal sensitivity and considering others.	• Initiates structure.<br>• Allows autonomy.<br>• Offers support.<br>• Maintains interpersonal sensitivity.
135	Abdulla et al. (2023)	Leadership development in academia in the UAE: creating a community of	• Emphasizes the idea of a cooperative learning environment helps the transfer of knowledge	• Acknowledges and is aware of

		learning.	vertically and horizontally.	their/team needs.<br>• Facilitates information sharing.
136	DeChurch and Marks (2006)	Leadership in multiteam systems	• Emphasizes that leaders must be able to monitor team information, as well as touches upon the importance of goals.	• Initiates structure.<br>• Acknowledges and is aware of their/team needs.
137	Bach and Sulíková (2021)	Leadership in the context of a NewWorld: digital leadership and industry 4.0	• Discusses goal setting.	• Initiates structure.
138	Wotruba (2016)	Leadership team coaching: A trust-based coaching relationship	• Emphasizes importance of creating a psychologically safe environment alongside picking up team members' feelings.	• Acknowledges and is aware of their/team needs.<br>• Facilitates information sharing.
139	Twehous et al. (1991)	Leadership training – The key to an effective program	• Discusses the importance of interpersonal relationships.	• Maintains interpersonal sensitivity.
140	Hutchins (2022)	Leading by nature for flourishing future-fit business: Embracing an OD in the service of life-affirming futures	• Discusses transformative learning and learning to iterate forward.	• Engages in reflective practice.
141	Harris and Cullen (2008)	Learner-centered leadership: An agenda for action.	• Discusses learning theory and the importance of considering ways in which team members can learn from each other.	• Offers support.<br>• Organizes team information.
142	Reime et al. (2017)	Learning by viewing versus learning by doing: A comparative study of observer and participant experiences during an interprofessional simulation training.	• Discusses a simulation-based training that helped learners develop skills in closed-loop communication, team cooperation, and more.	• Maintains interpersonal sensitivity.<br>• Engages in open dialogue.

143	Gamble and Vaux (2014)	Learning leadership skills in practice through quality improvement.	• Emphasizes the importance of continuous learning.	• Engages in reflective practice.
144	Schneider (2023)	Leveling up video game industry leaders through applied OD principles	• Discusses the challenges of the video game industry and how organizational development principles apply, as well as the importance of strengthening team development and enhancing strategic communications.	• Offers support.
145	Debets et al. (2023)	Linking leadership development programs for physicians with organization-level outcomes: A realist review.	• Discusses how a safe environment enabled information sharing.	• Facilitates information sharing.
146	Wood and Gordon (2009)	Linking MBA learning and leadership coaching.	• Discusses how the coach can help predict problems and plan, and how role playing can help develop leaders.<br>• Emphasizes strategic self-awareness and the development of shared mental models.	• Acknowledges and is aware of their/team needs.<br>• Organizes team information.
147	Pradhan and Pradhan (2012)	Linking transformational leadership, karma-yoga, and contextual performance	• Discusses transformational leadership and how engaging with followers can raise motivation.	• Acknowledges and is aware of their/team needs.
148	Abrahamsen and Chroni (2021)	Longevity in elite coaching: Motives and maneuvers keeping them in the game	• Discusses fostering a positive environment that promoted success.	• Maintains interpersonal sensitivity.
149	Ballangrud et al. (2021)	Longitudinal team training program in a Norwegian surgical ward: A qualitative study of nurses' and physicians' experiences with implementation.	• Discusses how simulation allowed for a better safety culture and for people to feel safe learning from their mistakes.	• Offers support.<br>• Engages in open dialogue.<br>• Engages in reflective practice.
150	Sanko et al. (2012)	Man versus machine: The preferred modality.	• Discusses learning objectives such as improved communication skills and understanding the	• Acknowledges and is aware of

			dynamics of a team.<br>● Discussed debriefing.	their/team needs.<br>● Organizes team information.<br>● Maintains interpersonal sensitivity.<br>● Engages in reflective practice.
151	Moyer and Brown (2011)	Medical team training: Using simulation as a teaching strategy for group work	● Discussed psychological understanding of team errors and working together to achieve a common goal.<br>● Emphasizes how simulation can potentially help team members understand personal behaviors and learning paradigms within their team.	● Acknowledges and is aware of their/team needs.<br>● Maintains interpersonal sensitivity.<br>● Engages in reflective practice.
152	Dierdorff and Ellington (2012)	Members matter in team training: Multilevel and longitudinal relationships between goal orientation, self-regulation, and team outcomes	● Emphasizes how training requires self-regulation.	● Allows autonomy.
153	Lester et al. (2011)	Mentoring impact on leader efficacy development: A field experiment	● Emphasizes how leaders must think they can produce sufficient motivation to act and be aware of their team's learning orientation.<br>● Discusses how trust can facilitate knowledge transfer.	● Acknowledges and is aware of their/team needs.<br>● Facilitates information sharing.
154	Moore and Ahmed (2021)	Motivation and leadership in the healthcare environment	● Discusses how leaders in healthcare need to know how to motivate their employees and find their reason why.	● Offers support.<br>● Acknowledges and is aware of their/team needs.
155	Aripin et al. (2023)	Movers and shakers: Leadership	● Discusses how leaders should share their	● Offers support.

		attributes for a successful lean manufacturing implementation	knowledge and experiences with their team.	
156	Branda et al. (2018)	Optimizing huddle engagement through leadership and problem-solving within primary care: A study protocol for a cluster randomized trial.	• Discusses the need for psychological safety so people can feel as though they can express their opinions.	• Maintains interpersonal sensitivity.<br>• Facilitates information sharing.
157	Despins (2009)	Patient safety and collaboration of the intensive care unit team	• Emphasizes how when team members feel they are working in teams characterized by interpersonal trust and respect, they are more enthusiastic to participate in quality improvement efforts.	• Maintains interpersonal sensitivity.
158	Klipfel et al. (2014)	Patient safety improvement through in situ simulation interdisciplinary team training.	• Discusses respectful communication as a foundation for establishing partnerships between persons.	• Maintains interpersonal sensitivity.
159	Wisshak and Barth (2022)	Perceptions of accountability for the transfer of training by leadership trainers.	• Discusses how accountability can lead to greater effort and performance, as well as the importance of understanding one's goals.	• Initiates structure.<br>• Allows autonomy.
160	Longman et al. (2018)	Responsibility as a motivator for women to lead	• Discusses gender differences in leadership and the importance of sense of purpose.	• Initiates structure.<br>• Acknowledges and is aware of their/team needs.
161	Edwards et al. (2006)	Relationships among team ability composition, team mental models, and team performance.	• Discusses the importance of team mental models and accuracy.	• Organizes team information.
162	Qiu et al. (2021)	Research and countermeasures on leadership development of growth thinking model leaders in Chinese high technology enterprises.	• Emphasizes that to lead is to influence the behavior of others and the importance of experiential leadership learning.	• Initiates structure.<br>• Engages in reflective practice.

163	Fielder (1996)	Research on leadership selection and training: One view of the future	• Discusses interpersonal tensions between members.	• Maintains interpersonal sensitivity.
164	Parry and Sinha (2005)	Researching the trainability of transformational organizational leadership.	• Discusses goal setting theory and action-learning.	• Initiates structure.<br>• Engages in reflective practice.
165	Mullen and Kelloway (2009)	Safety leadership: A longitudinal study of the effects of transformational leadership on safety outcomes.	• Discusses goal leadership and how to develop personal goals, and the importance of challenging individuals.	• Initiates structure.<br>• Allows autonomy.
166	Gray et al. (2014)	Salutogenisis and coaching: Testing a proof of concept to develop a model for practitioners.	• Discusses how coaching can help with positive states by developing a sense of humanity.	• Maintains interpersonal sensitivity.
167	Goldsby et al. (2021)	Self-leadership: A four decade review of the literature and trainings	• Emphasizes the importance of allowing people to pursue their own work goals.	• Allows autonomy.
168	Krampitz et al. (2021)	Self-leadership: A meta-analytic review of intervention effects on leaders' capacities	• Discusses self-leadership and self-efficacy regarding one's goals.	• Initiates structure.
169	Flentje et al. (2016)	Simulating a patient's fall as a means to improve routine communication: Joint training for nursing and fifth-year medical students.	• Emphasizes the importance of recognizing potentials for conflict and how they can influence routine communication.	• Maintains interpersonal sensitivity.
170	Motola et al. (2013)	Simulation in healthcare education: A best evidence practical guide. AMEE Guide No. 82.	• Discusses the importance of having learners take responsibility for their own progress, the coordination of effort and adaptation to situational factors.<br>• The environment should be non-threatening to the learners.<br>• Debriefing is important to assess performance.	• Allows autonomy.<br>• Engages in reflective practice.
171	Galloway (2009)	Simulation techniques to bridge the	• Discusses debriefing and importance of	• Engages in

		gap between novice and competent healthcare professionals	constructive feedback.	reflective practice.
172	Marshall and Flanagan (2010)	Simulation-based education for building clinical teams.	• Emphasizes how communication, leadership, and group decision making are essential to determine the priorities and goals for the team.<br>• Discusses the importance of a positive training climate.	• Initiates structure.<br>• Facilitates information sharing.
173	Weaver et al. (2010)	Simulation-based team training at the sharp end: A qualitative study of simulation-based team training design, implementation, and evaluation in healthcare	• Discusses how simulation fosters effective learning by keeping people engaged.	• Engages in reflective practice.
174	Davidovitch et al. (2010)	Simulator-based team training to share resources in a matrix structure organization	• Discusses team knowledge and how it emerges because of interactions among team members.	• Organizes team information.
175	Arumugam and Linderman (2022)	Six Sigma and operational absorptive capacity: The role of project leader	• Discusses individual goal keepers and how knowledge moves through organizations.	• Initiates structure.<br>• Organizes team information.
176	Drennan and Richey (2012)	Skills-Based Leadership The First-Line Supervisor Part I.	• Emphasizes the importance of feedback for learning.	• Engages in reflective practice.
177	Massenberg et al. (2015)	Social support at the workplace, motivation to transfer and training transfer: A multilevel indirect effects model	• Emphasizes the importance of social support.	• Offers support.
178	Lucas and Turner (2023)	Spiraling the field: A dynamic model exploring reflective maturity, reflective capacity and the expanding reflective field.	• Discusses how psychological safety makes it easier to reflect.	• Facilitates information sharing.
179	Greer (2016)	Step up and be a leader	• Discusses qualities of an effective leader.	• Maintains

			(focuses on interpersonal competencies) and how promoting open communication and removing silos is important.	interpersonal sensitivity.<br>● Engages in open dialogue.
180	Garza and Salcedo (2021)	Strategies for fostering emotional intelligence among organizational leaders.	● Discusses how it is important to emphasize the why of the goal, provide opportunities for discussion, and provide feedback.	● Initiates structure.<br>● Engages in open dialogue.
181	Zapalska et al. (2015)	Strategies for self-leadership development: An example of the US Coast Guard Academy	● Discusses the importance of having clearly defined goals and emphasizes high-quality feedback.<br>● Discusses individuals who engage with new knowledge by sharing ideas and opinions.	● Initiates structure.<br>● Facilitates information sharing.
182	Rhodes and Hands (2020)	Student engagement for enhancing mentoring and leadership development in adult basic education and literacy programs	● Discusses social capital and how it can help reinforce learning.	● Maintains interpersonal sensitivity.
183	Philip and Aguilar (2022)	Student perceptions of leadership skills necessary for digital transformation	● Discusses the importance of goal setting.	● Initiates structure.
184	O'Bannon et al. (2010)	Successful leadership development for women STEM faculty	● Describes a program that allowed participants to explore their own leadership style and self-select their training.	● Allows autonomy.
185	Kuntz and Livingstone (2020)	Surveying women leaders' career trajectories: Implications for leadership development in New Zealand organizations.	● Goal setting is important.<br>● Discusses the importance of psychological safety and vicarious learning.	● Initiates structure.<br>● Facilitates information sharing.
186	Sebastian and Hühn (2024)	Sustainable leadership and hegelian self-awareness	● Discusses ethical leadership and the need to be aware of social complexity.	● Acknowledges and is aware of their/team needs.<br>● Maintains interpersonal

				sensitivity.
187	Wallin et al. (2007)	Target-focused medical emergency team training using a human patient simulator: Effects on behavior and attitude	• Taught knowledge of the environment via simulation.	• Engages in reflective practice.
188	Warde et al. (2020)	Teaching primary care teamwork: A conceptual model of primary care team performance.	• Discusses goal specification and clarifying aims as well as ground rules.<br>• Emphasizes a relationship-centered culture where respect and listening are valued.<br>• Team training approach.	• Initiates structure.<br>• Offers support.
189	Vito and Hanbridge (2020)	Teaching social work leadership and supervision: Lessons learned from on-campus and online formats	• Discusses a variety of assessments to help with learning, reflection paper was favored for value in learning.	• Engages in reflective practice.
190	Snyder (2009)	Teaching teams about teamwork: Preparation, practice, and performance review	• Discusses the need for students to practice their communication skills prior to real-time performance.	• Engages in reflective practice.
191	Lerner et al. (2009)	Teaching teamwork in medical education.	• The team needs to have willingness to cooperate with the shared goal set by the team.<br>• Clarifying issues helps, people need to understand what everyone is contributing and why.	• Initiates structure.<br>• Offers support.<br>• Engages in reflective practice.
192	Ghosh (2020)	Team coaches' experiences of coaching to develop conditions for shared leadership	• Emphasized how coaching can open up dialogue and establish a shared framework of knowledge, as well as reflection.	• Organizes team information.<br>• Facilitates information sharing.<br>• Engages in reflective practice.
193	Anderson et al.	Team coaching helps a leadership team	• Emphasizes how to create alignment in goals	• Maintains

	(2008)	drive cultural change at Caterpillar.	and foster relationships.	interpersonal sensitivity.<br>• Organizes team information.
194	Van Wyk et al. (2019)	Team coaching in the workplace: Critical success factors for implementation.	• Discusses what factors team coaching needs to be successful, with a focus on goals.<br>• Emphasizes how team coaching is a platform to create knowledge sharing.	• Initiates structure.<br>• Facilitates information sharing.
195	Hauser (2018)	Team Coaching Operating System (TCOS): The intersection of evidence-based research and gestalt principles.	• Discusses goal setting.<br>• Emphasizes how the coach can help the team reflect on their experiences.	• Initiates structure.<br>• Engages in reflective practice.
196	Pavlović (2021)	Team coaching psychology: Toward an integration of constructivist approaches	• Discusses building a shared vision around a goal.<br>• Emphasizes team coaching as an aspect of leadership.	• Initiates structure.<br>• Offers support.
197	Hastings and Pennington (2019)	Team Coaching: A thematic analysis of methods used by external coaches in a work domain.	• Discusses focusing on the task to help improve the motivation sphere.<br>• Emphasizes relational dynamics and systemic context of the team, and how external coaches can gather information on external circumstances to the team as well as the team itself.	• Acknowledges and is aware of their/team needs.
198	Traylor et al. (2020)	Team coaching: Three questions and a look ahead: A systematic literature review.	• Discusses a variety of team-related behaviors, from goal-oriented behavior, information sharing, to having goal commitment, as well as the need for interpersonal communication.<br>• Emphasizes knowledge integration as a key factor.	• Maintains interpersonal sensitivity.<br>• Facilitates knowledge sharing.<br>• Organizes team information.

199	Slay et al. (1998)	Team training for home-based blind rehabilitation.	• Discusses shared knowledge as a cornerstone of the teaming process.	• Organizes team information.
200	Lu et al. (2010)	Team training in China: Testing and applying the theory of cooperation and competition	• Emphasizes how motivation is enhanced when people know their goals.<br>• Discusses the importance of feedback to help people improve their work and strengthen their ongoing learning.	• Initiates structure<br>• Engages in reflective practice.
201	Salas et al. (2001)	Team training in the skies: Does crew resource management (CRM) training work?	• Discusses how CRM improves learning.	• Engages in reflective practice.
202	Zhang and Wang (2023)	Teammate conscientiousness diversity depletes team cohesion: The mediating effect of intra-team trust and the moderating effect of team coaching	• Discusses how different personality traits may affect team cohesion but emphasizes the importance of trust to build knowledge.	• Maintains interpersonal sensitivity.<br>• Facilitates knowledge sharing.<br>• Organizes team information.
203	Salas et al. (2015)	Teams in space exploration: A new frontier for the science of team effectiveness	• Discusses the importance of team cohesion and the need for further understanding it.	• Facilitates information sharing.
204	Baker et al. (2006)	Teamwork as an essential component of high-reliability organizations	• Emphasizes the importance and willingness to cooperate for a shared goal as well as allowing members to practice what they are learning and providing feedback.	• Engages in open dialogue.<br>• Engages in reflective practice.
205	Ismail et al. (2017)	The administration of leadership training programs enhance the trainees' motivation to learn	• Emphasizes how positive behavior may lead to the support and maintenance of the organizational strategy and goals.	• Facilitates information sharing.

206	Keller and Olson (2000)	The advisability of outdoor leadership training: Caveat emptor	• Discusses fidelity of training circumstances.	• Engages in reflective practice.
207	Conger (1993)	The brave new world of leadership training.	• Discusses a need to clarify the team goal, as well as having the leader be the role model.<br>• Discusses action learning and shared reflections.	• Initiates structure.<br>• Offers support.<br>• Maintains interpersonal sensitivity.
208	Hewison (2004)	The crisis of cultural leadership in Britain	• Discusses creating a vision and setting targets.	• Initiates structure.
209	Silla et al. (2020)	The cross-level relationship between organizational trust in leadership and job satisfaction	• Emphasizes the importance of setting group goals and aligning individual goals with a shared mission.	• Initiates structure.<br>• Allows autonomy.
210	Salas et al. (2000)	The design and delivery of crew resource management training: Exploiting available resources.	• Discusses how declarative knowledge becomes procedural knowledge through practice or rehearsal.	• Engages in open dialogue.<br>• Engages in reflective practice.
211	Fiedler (1972)	The effects of leadership training and experience: A contingency model interpretation	• Discusses the contingency model that postulates that group performance depends on situational favorableness.	• Offers support.
212	Grulke et al. (2001)	The effects of physical environment on engineering team performance: A case study	• Discusses the importance of having an environment that supports teamwork.	• Offers support.
213	Fulmer (1997)	The evolving paradigm of leadership development	• Emphasizes that the purpose of leadership is to provide knowledge when it is required.	• Offers support.

214	Carr and Peters (2013)	The experience of team coaching: A dual case study.	• Discusses how the coach can support team members to refresh motivation and the need for an effective team contract.<br>• Discusses focusing on the structure of the team.<br>• Emphasizes factors such as information sharing.	• Initiates structure<br>• Offers support.<br>• Organizes team information.
215	Natale and Diamante (2005)	The five stages of executive coaching: Better process makes better practice	• Discusses executive coaching and how it may help manage stress, interpersonal relationships, and improve performance.	• Maintains interpersonal sensitivity.
216	Tompson and Tompson (2013)	The focus of leadership development in MNCs	• Discusses a model of holistic leadership that emphasizes understanding surroundings.	• Acknowledges and is aware of their/team needs.
217	de Vries (2014)	The group coaching conundrum	• Discusses how learning can occur and how constructing our self-regard through positive appraisal of others is important for learning.	• Facilitates information sharing.<br>• Engages in reflective practice.
218	Tremaine (2016)	The high-flying leadership qualities: What matters the most?	• Emphasizes that a leader must be able to communicate vision/purpose to the organization to understand goals.<br>• Emphasizes that leaders must be able to share knowledge.	• Initiates structure.<br>• Facilitates information sharing.
219	Cannon-Bowers et al. (1998)	The impact of cross-training and workload on team functioning: A replication and extension of initial findings	• Discusses how cross-training can help team performance and how teams must rely on their own knowledge when performing.	• Organizes team information.

220	Salamon et al. (2021)	The interplay between the level of voluntary participation and supervisor support on trainee motivation and transfer	• Emphasizes supervisor support and how this can facilitate trainee motivation.<br>• Discusses the importance of acknowledging other perspectives and providing feedback.	• Offers support.<br>• Engages in open dialogue.<br>• Engages in reflective practice.
221	Saravo et al. (2017)	The need for strong clinical leaders – Transformational and transactional leadership as a framework for resident leadership training.	• Discusses the importance of feedback.	• Engages in reflective practice.
222	Lippitt (1943)	The psychodrama in leadership training	• Discusses interpersonal relationship skills for leadership roles.	• Maintains interpersonal sensitivity.
223	Deluga (1995)	The relation between trust in the supervisor and subordinate organizational citizenship behavior	• Discusses the importance of interpersonal trust for effectiveness and highlights organizational citizenship behaviors.	• Facilitates information sharing.
224	Brion (2020)	The role of culture in the transfer of training.	• Discusses factors that affect knowledge transfer with an emphasis on how culture can affect this.	• Acknowledges and is aware of their/team needs.<br>• Maintains interpersonal sensitivity.
225	Tudor Car et al. (2018)	The role of eLearning in health management and leadership capacity building in health system: A systematic review.	• Discusses how attitude towards learning is vital for success.	• Facilitates information sharing.
226	Mbokota and Reid (2022)	The role of group coaching in developing leadership effectiveness in a business school leadership development programme.	• Discusses the importance of psychological safety and providing individuals with their needs and feedback.	• Acknowledges and is aware of their/team needs.<br>• Engages in reflective

				practice.
227	Stiehl et al. (2015)	The role of motivation to lead for leadership training effectiveness	• Discusses circumstances around training effectiveness and the need to accept new challenges and mobilize to reach organizational goals.	• Initiates structure.
228	Yaghi and Bates (2020)	The role of supervisor and peer support in training transfer in institutions of higher education	• Discusses how the work environment may affect motivation, and the importance of opportunities to practice, goals and incentives, and performance feedback.	• Initiates structure.<br>• Engages in reflective practice.
229	Tabernero et al. (2009)	The role of task-oriented versus relationship-oriented leadership on normative contract and group performance	• Looks at leader consideration and initiating structure, both of which had effects on performance.	• Initiates structure.<br>• Offers support.
230	Stout et al. (1997)	The role of trainee knowledge structures in aviation team environments	• Training improved knowledge structures of the team.	• Facilitates information sharing.
231	Terblanche and Erasmus (2018)	The use of organizational network analysis as a diagnostic tool during team coaching	• Discusses goal setting and benefits of team coaching behaviors, such as self-regulation, development of trust within the group, and more.	• Initiates structure.<br>• Allows autonomy.<br>• Maintains interpersonal sensitivity.
232	Turner and Parodi (2012)	Theoretically-driven infrastructure for supporting health care teams training at a military treatment facility.	• Discusses debriefs and the importance of giving feedback for performance	• Engages in reflective practice.
233	Shuffler et al. (2011)	There's a science for that: Team development interventions in organizations	• Discusses the importance of goal clarity and goal setting to reduce conflict and what team training can accomplish for teams.	• Initiates structure.<br>• Engages in reflective

			• Emphasizes that team training allows teams to receive feedback.	practice.
234	Neck and Manz (1996)	Thought self-leadership: The impact of mental strategies training on employee cognition, behavior, and affect.	• Discusses self-leadership and the training's ability to increase positive affect and decrease negative affect, among other results.	• Acknowledges and is aware of their/team needs.
235	Rosen et al. (2010)	Tools for evaluating team performance in simulation-based training.	• Emphasizes the importance of evaluation to be able to accurately measure training's results.	• Engages in reflective practice.
236	Tannenbaum and Yukl (1992)	Training and development in work organizations	• Emphasizes setting goals.<br>• Discusses pre-training expectations and how they can help better commitment.	• Initiates structure.
237	Vinhateiro et al. (2012)	Training for the next generation of coastal management practitioners.	• Emphasizes the importance of interpersonal skills for managers.	• Maintains interpersonal sensitivity.
238	Valdiri et al. (2015)	Training forward surgical teams for deployment: The US Army trauma training center	• Discusses TeamSTEPPS amongst other programs and the importance of hands-on-learning for performance.	• Emphasizes reflective practice.
239	Schulman et al. (2010)	Training forward surgical teams: Do military-civilian collaborations work	• Discusses goal setting.<br>• Emphasizes the importance of considering the learning environment for these collaborations to work.	• Initiates structure.<br>• Acknowledges and is aware of their/team needs.
240	Wisborg et al. (2006)	Training multiprofessional trauma teams in Norwegian hospitals using simple and low-cost local simulations	• Discusses communication fails and pitfalls as well as the importance of the setting for facilitating change in hospitals.	• Engages in open dialogue.<br>• Facilitates information sharing.
241	Blume et al. (2010)	Transfer of training: A meta-analytic review.	• Focused on training transfer and discussed precursors at large that affect training.	• Initiates structure.

			Motivation prior to training affects transfer.	
242	Vincent et al. (2015)	Triggers, timing and type: Exploring developmental readiness and the experience of consciousness transformation in graduates of Australian leadership programmes	• Discusses a variety of interpersonal traits.	• Maintains interpersonal sensitivity.
243	Patwell et al. (2019)	Triple impact coaching: Use of the self in the coaching process reflecting on the past present and future	• Discusses intentionality for learning as well as the importance of feedback.	• Engages in reflective practice.
244	Schmutz et al. (2018)	Twelve tips for integrating team reflexivity into your simulation-based team training.	• Emphasizes the importance of team reflexivity.	• Engages in reflective practice.
245	Smith and Gilbert (2013)	Two coaches operating as a 'learning pair'.	• Discusses the experience of two coaches working as a pair and the benefits of doing so. Moreover, they discuss their preparedness in being a learning team and the importance of being open and taking advice and constructive criticism, among others.	• Engages in open dialogue.<br>• Engages in reflective practice.
246	Alizadeh et al. (2017)	Uncover it, students would learn leadership from Team-Based Learning (TBL): The effect of guided reflection and feedback.	• Emphasizes the importance of reflection and feedback and finds that this does matter for shared leadership capacity.	• Engages in reflective practice.
247	Bayer et al. (2017)	Understanding health policy leaders' training needs	• Discusses the knowledge elements needed for health policy leaders which, overall, involves being aware of internal and external policies surrounding the area.	• Acknowledges and is aware of their/team needs.
248	Saurbier (2014)	Using infographies as an integrative higher-order skill development assignment in undergraduate leadership instruction	• Discusses the development of a worksheet for undergraduate leadership capacity and its ability to improve higher-order thinking skills and time management.	• Allows autonomy.

249	Grossman et al. (2013)	Using instructional features to enhance demonstration-based training in management education	• Discusses goal setting.<br>• Discusses and introduces demonstration-based training (DBT) and its components, trainee characteristics, situational variables, learning processes, and learning outcomes.	• Initiates structure.<br>• Acknowledges and is aware of their/team needs.
250	Cerrone et al. (2017)	Using Objective Structured Teaching Encounters (OSTEs) to prepare chief residents to be emotionally intelligent leaders.	• Discusses emotional intelligence and the program's ability to reinforce both interpersonal and communication skills.	• Maintains interpersonal sensitivity.
251	Ulrich and Crider (2017)	Using teams to improve and performance.	• Discusses how to create teams, select teams, and the role of the team leader and member.<br>• Defines the qualities of a high-performing teams, such as having a clear goal, having a strong sense of collective identity, continuing collaboration, and more.	• Initiates structure.<br>• Offers support.
252	Murphy et al. (2019)	Using theories of behavior change to transition multidisciplinary trauma team training from the training environment to clinical practice.	• Discusses a program that involves identifying the problem, identifying facilitators and barriers, possible solutions, and implementation options.	• Acknowledges and is aware of their/team needs.
253	Stevens et al. (2007)	Utility of treatment implementation methods in clinical trial with rehabilitation teams	• Introduces intervention types that involve interactive workshops, written documents incorporating feedback, structured phone calls, and so forth.	• Engages in reflective practice.
254	Jacobson and Paynter (2019)	Verdict pending: Understanding leadership role identity for North Carolina judges and lawyers	• Discusses identity in lawyers for leadership such as self-constructed identity and perceived role identity.	• Acknowledges and is aware of their/team needs.
255	Lawrence and Whyte (2017)	What do experienced team coaches do?: Current practice in Australia and New Zealand	• Discusses variety in team coaching approaches, such as some focusing on the task and others on interpersonal relationships.<br>• Overall, the majority of team coaches start with a task strategy that helps everyone envision the mission at hand.	• Initiates structure.

256	Lieff et al. (2016)	What do I do? Developing a competency inventory for postgraduate (residency) program directors.	• Introduces competencies for residency program directors, includes communication and relationship management, leadership, professionalism, environmental engagement, and others.	• Maintains interpersonal sensitivity.<br>• Acknowledges and is aware of their/team needs.
257	Graves et al. (2021)	What do the experiences of team coaches tell us about the essential elements of team coaching?	• Introduces perspectives on coaching the team leader which include elements such as engaging the leader, making an agreement with them.<br>• Discusses the roles adopted by a team coach such as bringing in outside perspectives, transferring skills, facilitation, and helping with flow.	• Initiates structure.<br>• Acknowledges and is aware of their/team needs.
258	Krumm et al. (2016)	What does it take to be a virtual team player? The knowledge, skills, abilities, and other characteristics required in virtual teams.	• Discusses setting clear goals and the importance of allowing autonomous work and acting on own initiative.	• Initiates structure.<br>• Allows autonomy.
259	MacKie (2015)	Who sees change after leadership coaching? An analysis of impact by rater level and self-other alignment on multi-source feedback.	• Discusses how transformational leadership can change after coaching.<br>• Emphasizes the importance of manager support for coaching to be successful as well as goal alignment.	• Initiates structure.<br>• Offers support.
260	Kaufman and Grace (2011)	Women in grassroots leadership: Barriers and biases experienced in a membership organization dominated by men	• Discusses barriers women encounter when leading.	• Offers support
261	George and Sreedharan (2023)	Work life balance and transformational leadership as predictors of employee job satisfaction	• Transformational leadership has a positive effect on employees by supporting them in their job.	• Offers support.
262	O'Neill and Lamm (2000)	Working as a learning coach team in action learning.	• Discusses a learning coach approach and how a coach can help facilitate distinct processes such as achieving consensus.	• Offers support.<br>• Engages in open dialogue.
